# Prevalence and risk factors of posttraumatic stress symptoms among Internally Displaced Christian couples in Erbil, Iraq

**DOI:** 10.3389/fpubh.2023.1129031

**Published:** 2023-03-23

**Authors:** Sana Rofo, Lina Gelyana, Stefania Moramarco, Luma H. H. Alhanabadi, Faiq B. Basa, Antonio Dellagiulia, Leonardo Emberti Gialloreti

**Affiliations:** ^1^Department of Psychology, Salesian University of Rome, Rome, Italy; ^2^Department of Biomedicine and Prevention, University of Rome Tor Vergata, Rome, Italy; ^3^Department of Primary Health Care, Preventive Health Affairs Directorate, Duhok, Iraq; ^4^Internal Medicine Department, Rizgary Teaching Hospital, Erbil, Iraq

**Keywords:** post-traumatic stress disorder, Internally Displaced People, mental health, minorities, war, Iraq

## Abstract

**Background:**

Research about the impact of war and displacement experiences on the mental health of Internally Displaced People (IDPs) has recently grown. However, a limited number of studies focus on minorities. The objective of the present preliminary study was to estimate the prevalence of posttraumatic stress symptoms (PTSSs) among IDPs who live outside camps and belong to the Christian minority in Iraq, and to identify possible predictors.

**Methods:**

Overall, 108 internally displaced Christians (54 married couples) participated in the study. Traumatic events and PTSSs were assessed using the Harvard Trauma Questionnaire. Multivariable linear regression models were used to investigate possible predictors of PTSSs. Multivariable logistic regression models have been developed to estimate the odds of presenting PTSSs.

**Results:**

Results demonstrated high rates of trauma exposure, with all participants having experienced at least three traumatic events. The estimated prevalence of PTSSs was 20.3%. A low economic status, the number of traumatic events, and a second experience of displacement were associated with increased PTSSs. Five traumatic events were identified as the main predictors of PTSSs.

**Conclusion:**

Findings from the current preliminary study indicated the impact of war-related traumatic events on IDPs' mental health and the negative effects of post-displacement experiences. These findings may have important implications for setting up psychosocial interventions, as well as for further promoting physical and mental health services among these populations.

## 1. Introduction

Historically, Iraq is one of the Arab countries that has been highly affected by wars. It has been considered a highly traumatic environment (1), as it has been consistently exposed to different political conflicts during the last four decades, including the Iraqi-Iran war (1980–1988), the Anfal Campaign (1986–1990), the first (1990–1991) and second Gulf war (2003), and the war against the so-called Islamic State of Iraq and Syria (ISIS) (2014–2017) (2).

All these events resulted in vast population displacements, especially in the Iraqi Kurdistan Region (KRI), an autonomous region of northern Iraq and a relatively safe area in which to seek refuge (3). According to the United Nations High Commissioner for Refugees (UNHCR), more than 3 million people were displaced across the country due to the 2014 conflict (4). Based on the most recent report of the International Organization for Migration (IOM), in 2022, 1,177,234 Internally Displaced People (IDPs) are living in Iraq (5), with the KRI hosting the largest number of protracted IDPs (30%) (6).

The ongoing conflict has resulted in a high-risk population for developing mental health disorders such as post-traumatic stress disorder (PTSD) and depression (7). As a vulnerable population, IDPs suffer from a high rate of mental health disorders due to their exposure to different traumatic events before and during the migration or displacement period (8).

Recently, researchers have been focusing on the risk factors for PTSD among refugees and IDPs, investigating not only those related to pre-displacement but also post-displacement (9–12). The displacement experiences or post-displacement difficulties are regarded as significant risk factors for developing mental health disorders such as PTSD, so addressing these factors may aid in the recovery from the traumatic experiences as well as the effectiveness of mental health care programs (13, 14).

Christians are part of the IDPs populations in Iraq. Before the 2003 war, Christians were estimated to be ~1.5 million (15). In 2014, the so-called ISIS attacked their villages in Nineveh plain, displacing the entire community in the KRI and neighboring countries, with many Christians forced to flee multiple times. Hence, according to local Church estimations, the number of Christians has dwindled over the past 17 years to 250,000 (15).

In recent years, the research highlighted that the prevalence of PTSD among IDPs in the country has rapidly grown (11, 16–18). However, there are still limited studies on the prevalence of PTSD among minorities, particularly Christians. In Iraq, only one previous study has examined the prevalence of PTSD among different religious groups (19). However, the study mentioned above was conducted in refugee camps; to our knowledge, no investigations have been conducted among Internally Displaced Christians (IDC) living outside camps in Iraq. One characteristic of the Christian IDPs was that most lived in private houses, not camps. Therefore, we were interested in examining the impact of traumatic war events on the mental health of IDPs living in apartments rather than camps. Thus, the current preliminary study aimed to investigate the prevalence of posttraumatic stress symptoms (PTSSs) (20) among Internally displaced Christians living in KRI apartments and determine their possible predictors.

## 2. Methods

### 2.1. Setting

In 2005, Iraq's Constitution recognized the Kurdistan Regional Government (KRG) A division of the Autonomous Region of Kurdistan into four governorates is currently in place: Duhok, Erbil, Sulaymaniya, and Halabja. Erbil is the capital of the KRI and the home of the governing bodies of the KRG. The KRI hosts a melting pot of populations consisting of Kurds, Assyrians, Chaldeans, Turkmen, Armenians, Yazidis, Arabs, and other minorities.

### 2.2. Sample

The study's convenience sample was drawn from a population of Christian families seeking assistance (housing and health care needs) from the Chaldean Catholic Diocese office supporting displaced Christians in Erbil. The inclusion criteria for the current study were: (a) having fled to Erbil from the areas invaded by ISIS; (b) being displaced in Erbil after 2014; (c) being parental couples. The current study included all adult couples who consented and volunteered to participate. There was no predetermined sample size. At the time of data collection, they lived in small, fully furnished apartments with access to primary services such as running water and electricity.

### 2.3. Questionnaire

Data relevant to the investigation were collected using a self-administered *ad hoc* questionnaire, which consisted of two parts:

- The socio-demographic section included basic demographic variables (age, gender, education level, economic status, and employment). Regarding economic status, participants were asked to put themselves in a pre-defined category: low, medium, and high. A specific question was asked to measure employment, i.e., “Do you have a job right now?”. Displacement-related questions, such as the city from which they fled and the frequency of displacement, were also included in this section.
- The Harvard Trauma Questionnaire (HTQ)—a self–report checklist designed by the Harvard Program for Refugee Trauma (HPRT)—examines traumatic events, torture, and posttraumatic stress symptoms (PTSSs). For the present study, the Iraqi version in the Arabic language, adapted by Shoeb et al. (21), was chosen. The HTQ consists of five parts (Trauma Events - Part I, Personal Description–Part II,–Head Injury- Part III, Trauma Symptoms- Part IV, Torture History- Part V), of which only two (I, IV) were used in the present study. Part I evaluates the number and types of potentially traumatic events (PTE); it consists of 43 items with a binary yes or no response. Part IV contains 45 items, but for the purpose of the current study, we used only the first 16 items that assess PTSSs according to the DSM-IV. Symptoms are rated on a four-point Likert scale from 1 (not at all) to 4 (extremely), and a sum score is the mean of the 16 items (the sum score of the 16 items divided by 16). A score above 2.5 (mean score>2.5) indicates a likelihood of clinical PTSD (21).

### 2.4. Data collection

The current study was conducted in April 2019 at the participants' residences. All the participants provided informed consent after being informed about the study's objectives. All couples agreed to participate in the study. However, participants were free to withdraw from the study at any time. During the interview, participants were informed about the questionnaires and answer options, then they were asked to complete the questionnaires independently. However, if they had any questions or doubts about the questionnaire, they could consult the interviewer. The interviews were conducted by two local psychologists trained in using the questionnaire.

The study protocol was approved by the Ethics committee of the Pontifical Salesian University, Rome, Italy (reference number CSF503).

### 2.5. Statistical analysis

Descriptive statistics were used to illustrate the socio-demographic characteristics of the sample and describe the severity of traumatic events and PTSSs. For the Harvard Trauma Questionnaire, Part IV, the internal consistency of the current sample was calculated: Cronbach's α was 0.88.

Bivariate Spearman's correlations were applied to estimate the association between the level of trauma exposure and PTS symptoms. Multivariable linear regression models were used to investigate possible predictors of PTS symptoms (outcome variable). Demographic variables (age, gender, education level, economic status, employment status, and number of children), number of traumatic events, and frequency of displacements experienced were included as independent variables. Univariable and multivariable logistic regression models have been developed to estimate the odds of PTSSs (categorical outcome variable) in relation to possible predictive variables. Each traumatic event was analyzed through a univariate logistic regression considering PTSSs as the outcome variable. Those events which yielded a statistically significant result at the univariate regression were included in the subsequent multivariable logistic regression. An alpha level of 0.05 was used for all statistical analyses. Unless otherwise specified, results are presented as mean±SD. All statistical analyses were performed using SPSS v.26.0 (IBM Corp., Armonk, NY, USA).

## 3. Results

A total of 108 displaced Christians (54 married couples) were included in this study. Three couples were excluded from the analyses due to missing data. As shown in Table 1, 46.3% of the participants were in the age group 40–49 years, with the number of children in each couple ranging from 1 to 7 (Mean: 3.17±1.14). According to the place of residence, 40.7 % of the individuals came from Qaraqosh, the largest city of the Nineveh Plain. In terms of educational level, 37% held a bachelor's degree. Overall, 52.8% were working. Among males, 75.9% had a job. As regards economic status, 69.4% reported lower-middle incomes. Regarding the frequency of the displacement experience, 32.4% had been displaced for the second time. Further socio-demographic characteristics of the participants are reported in Table 1.

**Table 1:** Socio-demographic characteristics of the participants.

		**Total (*n* = 108)**	**Male (*n* = 54)**	**Female (*n* = 54)**
Gender	Male	54 (50%)		
	Female	54 (50%)		
Age	30–39	29 (26.9%)	5 (9.3%)	24 (44.4%)
	40–49	49 (45.4%)	24 (44.4%)	25 (46.3%)
	50 and over	30 (27.8%)	25 (46.3%)	5 (9.3%)
Educational level	Primary school	29 (26.9%)	12 (22.2%)	17 (31.5%)
	Secondary school	22 (20.4%)	12 (22.2%)	10 (18.5%)
	High school	12 (11.1%)	3 (5.6%)	9 (16.7%)
	Bachelor	40 (37.0%)	24 (44.4%)	16 (29.6%)
	Master	5 (4.6%)	3 (5.6%)	2 (3.7%)
Employment	Yes	57 (52.8%)	41 (75.9%)	16 (29.6%)
	No	51 (47.2%)	12 (24.1%)	38 (70.4%)
Economic status	Low	7 (13.0%)		
	Middle	37 (68.5%)		
	High	10 (18.5%)		
Number of children	1	3 (5.6%)		
	2	12 (22.2%)		
	3	19 (35.2%)		
	4	15 (27.8%)		
	5	4 (7.4%)		
	7	1 (1.9%)		
Frequency of displacement experience^*^	1	36 (66.7%)		
	2	18 (33.3%)		
Hometown	Mosul	16 (29.6%)		
	Qaraqosh	22 (40.7%)		
	Karmles	7 (13.0%)		
	Bartela	9 (16.7%)		

* Number of times the person has been displaced.

The estimated prevalence of PTSSs among participants was examined by applying the HTQ cut-off (part IV). A mean score over 2.5 was reported by 20.3% of the participants, indicating a likelihood of clinical PTSD; half of those (*n* = 11) with suggestive PTSD came from Mosul City, which was the largest town occupied by ISIS.

**Figure 1:**
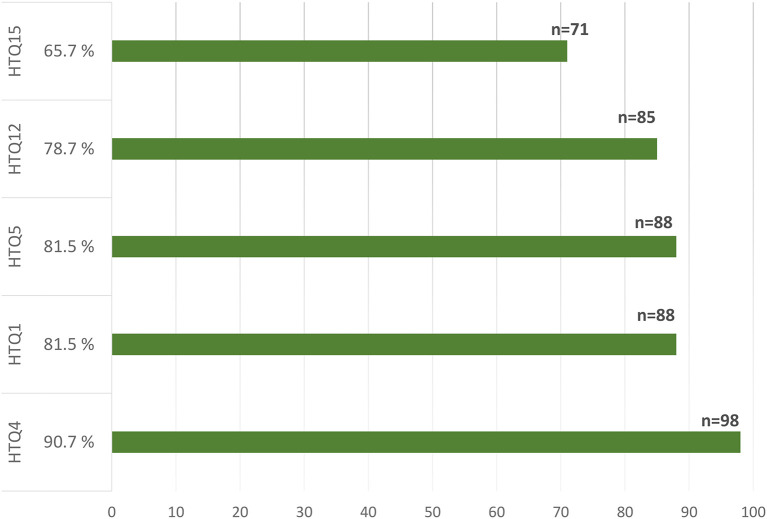
HTQ1: “oppressed because of ethnicity, religion, or sect”; HTQ4: “property looted, confiscated, or destroyed”; HTQ5: “forced to leave hometown and settle in a different part of the country with minimal services”; HTQ12: “witnessed the desecration or destruction of religious shrines or places of religious instruction”; HTQ15: “witnessed the shelling, burning, or razing of residential areas or marshlands.”

Participants reported having experienced between 3 and 24 traumatic events (Mean: 10.9 ± 4.84; Median: 10.5). Males experienced a mean of 11.7 ± 5.3 traumatic events (Median: 12.0), while females experienced an average of 10.2 ± 4.3 traumatic events (Median: 9.0). As shown in Figure 1, the most frequently reported traumatic events were, respectively: “property looted, confiscated, or destroyed”—HTQ4 (*n* = 98; 90.7%), “forced to leave hometown and settle in a different part of the country with minimal services”- HTQ5 (*n* = 88; 81.5%), “oppressed because of ethnicity, religion, or sect” -HTQ1 (*n* = 88; 81.5%), “witnessed the desecration or destruction of religious shrines or places of religious instruction” – HTQ12 (n.=85; 78.7%), “witnessed the shelling, burning, or razing of residential areas or marshlands”—HTQ15 (*n* = 71; 67.6%). The frequency of all experienced traumatic events is presented in Supplementary File 1. A significant positive correlation was found between the total number of traumatic events and PTSSs (*r* = 0.41; *p* ≤ *0.001*).

Multivariable linear regression was used to investigate the predictors of PTSSs. Socio-demographic variables, the number of traumatic events, and the frequency of displacement experiences were included in the model. A significant regression equation was found (F_(8, 99)_ = 6.29; *p* < 0.001), with an R^2^ of 0.34. Results indicated that a low economic status, the number of traumatic events, and a second experience of displacement were significantly associated with PTSSs. Gender, age, number of children, and educational level did not yield statistically significant results (Table 2).

**Table 2:** Predictors of posttraumatic stress symptoms (PTSSs).

**Variables**	**B**	**SE**	**β**	**t**	**p**	**95% CI**	
						**Lower**	**Upper**
Gender	0.113	0.119	0.102	0.948	0.346	−0.124	0.350
Age	0.089	0.077	0.118	1.153	0.252	−0.064	0.242
Educational level	−0.008	0.040	−0.019	−0.201	0.841	−0.086	0.070
Employment	0.166	0.116	0.149	1.432	0.155	−0.064	0.396
Economic status	−0.213	0.094	−0.211	−2.264	0.026	−0.400	−0.026
Number of children	−0.016	0.042	−0.033	−0.385	0.701	−0.100	0.068
Frequency of displacements experience	0.264	0.102	0.224	2.587	0.011	0.062	0.467
Number of traumatic events	0.041	0.011	0.353	3.639	< 0.001	0.019	0.063

Multivariable linear regression model. Outcome variable: Harvard Trauma Questionnaire (HTQ) score. B, Regression coefficient; SE, Standard Error; β, Adjusted Regression Coefficient.

We performed logistic regression models to examine which of the traumatic events determined a likelihood of clinical PTSD. Five traumatic events returned a statistically significant result at the univariate logistic regression, i.e., “present while someone searched for people or things in the home (HTQ2)”, “suffered ill health without access to medical care or medicine (HTQ7)”, “serious physical injury of a family member or friend from combat situation or landmine (HTQ20)”, “murder or violent death of a friend (HTQ31)”, “disappearance of a friend (HTQ35)”. These five events were included in the multivariable model. Table 3 reports in detail the HTQ variables feeding the statistical analysis.

**Table 3:** Predictors of possible presence of clinically relevant posttraumatic stress disorder.

	**Univariable**				**Multivariable**			
	**OR Exp (B)**	**Wald**	**p**	**95%CI**	**OR Exp (B)**	**Wald**	**p**	**95%CI**
Present while someone searched for people or things in the home (HTQ2)	4.471	8.934	0.003	1.675, 11.936	2.576	2.690	0.101	0.832, 7.982
Suffered ill health without access to medical care or medicine (HTQ7)	5.838	8.808	0.003	1.821, 18.719	3.909	4.533	0.033	1.114, 13.709
Serious physical injury of family member or friend from combat situation or landmine (HTQ20)	4.467	8.825	0.003	1.664, 11.990	2.347	2.095	0.148	0.739, 7.452
Murder or violent death of friend (HTQ31)	6.738	13.286	< 0.001	2.416, 18.797	2.937	2.456	0.117	0.736, 11.302
Disappearance of a friend (HTQ35)	5.267	7.562	0.006	1.612, 17.211	1.166	0.033	0.857	0.219, 6.202

Univariable and multivariable logistic regressions.

Outcome variable: dichotomized (≤ 2.5 vs. >2.5) Harvard Trauma Questionnaire (HTQ) score.

OR, odds ratio; Wald, Wald statistics; 95%CI, 95% confidence interval.

## 4. Discussion

The current study investigated the impact of traumatic events and forced displacement experiences on posttraumatic stress symptoms (PTSSs) among Internally Displaced Christian couples in the KRI. Results demonstrated high rates of trauma exposure, with all the participants having experienced at least three traumatic events.

According to a prior study, Christians were among the minority groups affected by traumatic events in Iraq (19). Also, in our study a high prevalence of PTSSs (over 20%) was found, although this data is lower compared to the 32% of PTSD reported by other authors (19). The difference between the two studies can be attributed to the methodological approaches, as well as the sample's characteristics and size, since Richa and colleagues conducted their research in refugee camps, with most of the participants having a low socio-demographic status.

Our finding is more in line with a systematic review on PTSD prevalence among Iraqi refugees in western countries, which ranged from 8 to 37.2% (1). It is also consistent with a previous study conducted on a sample of Iraqi IDPs, which reported a PTSD prevalence rate of 20.8% (16).

A secondary aim of our preliminary study was to investigate predictors of PTSSs. Previous scientific literature identified several risk factors among socio-demographic characteristics (10, 22, 23). In our study, participants reporting a low economic status were more likely to present with PTSSs. It can be speculated that participants with low economic status may have faced stressful experiences related to lack of food and difficulties getting access to health care services throughout the years. This finding is in line with prior studies reporting economic status as one of the risk factors for psychological distress or mental disorders (24–26), confirming that IDPs living outside camps might have even more socio-economic challenges than those residing inside the camps, due to a lack of continuous and systematized support and aid coverage from international and non-governmental organizations (19).

The present study revealed that being displaced for a second time was significantly associated with developing PTSSs. This finding aligns with the study among IDPs in South Ethiopia, which showed a positive association between displacement frequency and PTSD (27). This might be due to the fact that participants who experienced displacement for the second time have struggled with post-displacement living difficulties twice since these difficulties have been identified in previous studies as predictors of mental health disorders (10, 13, 28–31).

The number of traumatic events proved to be associated with PTSSs, indicating the effect of cumulative trauma exposure on developing PTSSs (11), confirming findings from previous studies among displaced populations (10, 22–24, 32, 33). When examining traumatic events that could have impacted on PTSSs, our findings showed that not having access to medical care in case of illness was a significant predictor of mental health symptoms. This finding is in line with a recent study of Ukrainian civilians involved in the war, which identified the lack of health insurance as a significant predictor of PTSD (12). Additionally, IDPs' health outcomes tend to be worse than those of conflict-affected populations due to a protracted lack of access to health services (34). A second traumatic event identified as a predictor of PTSSs was “being present while someone searched for people or things in the house”. Participants who experienced this event may have felt unsafe and feared for their and their families' lives. Previous studies have shown that feeling hazardous or having a low perceived feeling of safety is associated with the severity of PTSD (23, 26).

In accordance with findings from previous research that showed the impact of war-related traumas on PTSD, with specific regard to the death or injury of a relative/friend/loved one (12, 27, 33, 35, 36), in the present study, three war-related traumatic events, were identified as predictors of PTSSs: the murder or violent death of a friend, the disappearance of a friend, and the physical injury of a family member or friend in a combat situation. This is in line with a study including Rohingya refugees in Bangladesh, where mental health symptoms were more common in people who had experienced physical violence (20). Among the different possible traumatic events, “suffered ill health without access to medical care or medicine”, continued to yield a statistically significant increased risk of potential clinical PTSD, even after adjusting for other events, thus indicating the importance of not undervaluing this factor.

There are several limitations to be considered. The main limitation of this study was the limited representativeness of the sample since only Christians who were displaced near Erbil were included. It is, therefore, impossible to generalize the results to all IDPs. The study was performed as part of a humanitarian project focusing on treating individual cases, so its statistical power was limited due to the small number of participants. In addition, only married couples were included, which may have excluded a vulnerable population such us unmarried individuals and widows. Besides, other relevant mental health disorders beyond PTSSs, such as depression or anxiety, need to be further investigated, since the current study did not precisely screen these aspects (26, 37). Moreover, the questionnaire was self-administered, the study design was cross-sectional, and a previous assessment of PTSSs was not available. Even though HTQ has been shown to be a reliable instrument in non-western cultures and validated in several culturally different settings (38, 39), it is still a matter of discussion if the standard cut-off can be used to screen PTSSs across culturally diverse refugee populations (40). As a result, recall biases and inaccuracies in data collection cannot be excluded. This study identifies potential determinants of PTSSs but cannot establish any causality. Larger sample sizes should be used for future longitudinal studies, including also IDPs from other minorities and regions.

To the best of our knowledge, our research is among the first studies examining the prevalence and predictors of PTSSs among internally displaced minority groups in Iraq, primarily when referring to Christians living outside camps (19). Our findings demonstrated the impact of the war traumatic events and the negative effect of post-displacement difficulties on IDPs' mental health following war-related events. Besides presenting a preliminary analysis, the current study provides the basis for further investigations on mental health, PTSD, and difficulties among IDPs in Iraq. These steps are paramount for identifying the health priorities of these populations in a war-torn country, efficiently guiding health policies, and developing short- and long-term strategies and evidence-based decisions (41).

## Data availability statement

The raw data supporting the conclusions of this article will be made available by the authors, without undue reservation.

## Ethics statement

The study protocol was approved by the Ethics Committee of the Pontifical Salesian University, Rome, Italy (reference number CSF503). The patients/participants provided their written informed consent to participate in this study.

## Author contributions

SR, LG, and AD conceptualized and designed the overall project and the study and conceptualized the research. SR and LG conducted the interview and provided data collection with database generation. LA and FB coordinated the field activities, maintained contact with local authorities, and contributed to the writing process. SM and LE supported data analysis and finalized the manuscript. SR, LG, SM, and LE drafted the manuscript. All authors reviewed, provided input, suggested adjustments to the manuscript drafts, and approved the final text.
