# Supplementary material for: Prevalence and risk factors of posttraumatic stress symptoms among Internally Displaced Christian couples in Erbil, Iraq
Source: Front Public Health. 2023 Mar 23;11:1129031. doi: 10.3389/fpubh.2023.1129031 (PMC10076725; doi:10.3389/fpubh.2023.1129031)
Supplement: Supplementary file 1 [file Table_1.DOCX]

**Frequencies and Percentages of reported traumatic events (HTQs)**

| Traumatic events | Frequency | Percentage |
| --- | --- | --- |
| HTQ4  Property looted, confiscated, or destroyed | 98 | 90.7 % |
| HTQ5  Forced to leave your hometown and settle in a different part of the country with minimal services | 88 | 81.5 % |
| HTQ1  Oppressed because of ethnicity, religion, or sect | 88 | 81.5 % |
| HTQ12  Witnessed the desecration or destruction of religious shrines or places of religious instruction | 85 | 78.7 % |
| HTQ11  Lacked shelter | 71 | 65.7 % |
| HTQ15  Witnessed shelling, burning, or razing of residential areas or marshlands. | 71 | 65.7 % |
| HTQ22  Confined to home because of chaos and violence outside | 65 | 60.2 % |
| HTQ8  Suffered from lack of food or clean water | 57 | 52.8 % |
| HTQ7  Suffered ill health without access to medical care or medicine | 55 | 50.9 % |
| HTQ17  Exposed to combat situation (explosions, artillery fire, shelling) or landmine. | 53 | 49.1 % |
| HTQ9  Forced to flee your country | 51 | 47.2 % |
| HTQ10  Expelled from country based on ancestral origin, religion, or sect | 51 | 47.2 % |
| HTQ3  Searched. | 35 | 32.4 % |
| HTQ2  Present while someone searched for people or things in your home. | 34 | 31.5 % |
| HTQ20  Serious physical injury of family member or friend from combat situation or landmine. | 30 | 27.8 % |
| HTQ21  Witnessed rotting corpses | 26 | 24.1 % |
| HTQ13  Witnessed the arrest, torture, or execution of religious leaders or important members of tribe | 25 | 23.1 % |
| HTQ31  Murder or violent death of friend | 25 | 23.1 % |
| HTQ26  Witnessed murder | 20 | 18.5 % |
| HTQ23  Witnessed someone being physically harmed (beating, knifing, etc.) | 18 | 16.7 % |
| HTQ36  Family member (child, spouse, etc.) kidnapped or taken as a hostage | 18 | 16.7 % |
| HTQ30  Murder or violent death of family member (child, spouse, etc.) | 16 | 14.8 % |
| HTQ37  Friend kidnapped or taken as a hostage. | 16 | 14.8 % |
| HTQ35  Disappearance of a friend | 14 | 13.0 % |
| HTQ14  Witnessed mass execution of civilians | 11 | 10.2 % |
| HTQ38  Someone informed on you placing you and your family at risk of injury or death. | 11 | 10.2 % |
| HTQ34  Disappearance of a family member (child, spouse, etc.) | 10 | 9.3 % |
| HTQ43  Please specify any other situation that was very frightening or in which you felt your life was in danger | 10 | 9.3 % |
| HTQ25  Witnessed torture | 7 | 6.5 % |
| HTQ33  Forced to pay for bullet used to kill family member (child, spouse, etc.) | 7 | 6.5 % |
| HTQ18  Serious physical injury from combat situation or landmine. | 4 | 3.7 % |
| HTQ6  Imprisoned | 3 | 2.8 % |
| HTQ16  Witnessed chemical attacks on residential areas or . marshlands | 3 | 2.8 % |
| HTQ19  Used as a human shield | 2 | 1.9 % |
| HTQ27  Forced to inform on someone placing them at risk of injury or death | 2 | 1.9 % |
| HTQ42  Tortured (i.e., while in captivity you received deliberate and systematic infliction of physical and/or mental suffering) | 2 | 1.9 % |
| HTQ24  Witnessed sexual abuse or rape | 1 | 0.9 % |
| HTQ28  Forced to destroy someone’s property | 1 | 0.9 % |
| HTQ29  Forced to physically harm someone (beating, knifing, etc.) | 1 | 0.9 % |
| HTQ41  Sexually abused or raped (i.e., forced sexual activity) | 1 | 0.9 % |
